# Target engagement of the subgenual anterior cingulate cortex with transcranial temporal interference stimulation in major depressive disorder: a protocol for a randomized sham-controlled trial

**DOI:** 10.3389/fnins.2024.1390250

**Published:** 2024-08-29

**Authors:** Ilya Demchenko, Sumientra Rampersad, Abhishek Datta, Andreas Horn, Nathan W. Churchill, Sidney H. Kennedy, Sridhar Krishnan, Alice Rueda, Tom A. Schweizer, John D. Griffiths, Edward S. Boyden, Emiliano Santarnecchi, Venkat Bhat

**Affiliations:** ^1^Interventional Psychiatry Program, St. Michael’s Hospital – Unity Health Toronto, Toronto, ON, Canada; ^2^Institute of Medical Science, Temerty Faculty of Medicine, University of Toronto, Toronto, ON, Canada; ^3^Institute for Biomedical Engineering, Science and Technology (iBEST), Keenan Research Centre for Biomedical Science, St. Michael’s Hospital – Unity Health Toronto, Toronto, ON, Canada; ^4^Department of Physics, University of Massachusetts Boston, Boston, MA, United States; ^5^Department of Electrical and Computer Engineering, Northeastern University, Boston, MA, United States; ^6^Research and Development, Soterix Medical, Inc., Woodbridge, NJ, United States; ^7^Department of Biomedical Engineering, City College of New York, New York, NY, United States; ^8^Department of Neurology, Center for Brain Circuit Therapeutics, Brigham and Women’s Hospital and Harvard Medical School, Boston, MA, United States; ^9^Department of Neurosurgery & Center for NeuroTechnology and NeuroRecovery (CNTR), Department of Neurology, Massachusetts General Hospital and Harvard Medical School, Boston, MA, United States; ^10^Movement Disorder and Neuromodulation Unit, Department of Neurology, Charité – Universitätsmedizin Berlin, Corporate Member of Freie Universität Berlin and Humboldt – Universität zu Berlin, Berlin, Germany; ^11^Keenan Research Centre for Biomedical Science, St. Michael’s Hospital – Unity Health Toronto, Toronto, ON, Canada; ^12^Neuroscience Research Program, St. Michael’s Hospital – Unity Health Toronto, Toronto, ON, Canada; ^13^Department of Psychiatry, Temerty Faculty of Medicine, Toronto, ON, Canada; ^14^Department of Electrical, Computer, and Biomedical Engineering, Toronto Metropolitan University, Toronto, ON, Canada; ^15^Division of Neurosurgery, Department of Surgery, Temerty Faculty of Medicine, University of Toronto, Toronto, ON, Canada; ^16^Krembil Centre for Neuroinformatics, Centre for Addiction and Mental Health (CAMH), Toronto, ON, Canada; ^17^Department of Brain and Cognitive Sciences, Media Arts and Sciences, and Biological Engineering, McGovern Institute for Brain Research and Koch Institute for Integrative Cancer Research, Massachusetts Institute of Technology, Cambridge, MA, United States; ^18^Howard Hughes Medical Institute, Chevy Chase, MD, United States; ^19^Precision Neuroscience and Neuromodulation Program, Gordon Center for Medical Imaging, Department of Radiology, Massachusetts General Hospital and Harvard Medical School, Boston, MA, United States; ^20^Department of Neurology, Massachusetts General Hospital and Harvard Medical School, Boston, MA, United States

**Keywords:** mood disorders, brain stimulation, temporal interference, clinical trials, electric stimulation therapy, magnetic resonance imaging, electroencephalography, feasibility studies

## Abstract

**Background:**

Transcranial temporal interference stimulation (tTIS) is a new, emerging neurostimulation technology that utilizes two or more electric fields at specific frequencies to modulate the oscillations of neurons at a desired spatial location in the brain. The physics of tTIS offers the advantage of modulating deep brain structures in a non-invasive fashion and with minimal stimulation of the overlying cortex outside of a selected target. As such, tTIS can be effectively employed in the context of therapeutics for the psychiatric disease of disrupted brain connectivity, such as major depressive disorder (MDD). The subgenual anterior cingulate cortex (sgACC), a key brain center that regulates human emotions and influences negative emotional states, is a plausible target for tTIS in MDD based on reports of its successful neuromodulation with invasive deep brain stimulation.

**Methods:**

This pilot, single-site, double-blind, randomized, sham-controlled interventional clinical trial will be conducted at St. Michael’s Hospital **–** Unity Health Toronto in Toronto, ON, Canada. The primary objective is to demonstrate target engagement of the sgACC with 130 Hz tTIS using resting-state magnetic resonance imaging (MRI) techniques. The secondary objective is to estimate the therapeutic potential of tTIS for MDD by evaluating the change in clinical characteristics of participants and electrophysiological outcomes and providing feasibility and tolerability estimates for a large-scale efficacy trial. Thirty participants (18–65 years) with unipolar, non-psychotic MDD will be recruited and randomized to receive 10 sessions of 130 Hz tTIS or sham stimulation (*n* = 15 per arm). The trial includes a pre- vs. post-treatment 3T MRI scan of the brain, clinical evaluation, and electroencephalography (EEG) acquisition at rest and during the auditory mismatch negativity (MMN) paradigm.

**Discussion:**

This study is one of the first-ever clinical trials among patients with psychiatric disorders examining the therapeutic potential of repetitive tTIS and its neurobiological mechanisms. Data obtained from this trial will be used to optimize the tTIS approach and design a large-scale efficacy trial. Research in this area has the potential to provide a novel treatment option for individuals with MDD and circuitry-related disorders and may contribute to the process of obtaining regulatory approval for therapeutic applications of tTIS.

**Clinical Trial Registration:**

ClinicalTrials.gov, identifier NCT05295888.

## 1 Introduction

Major depressive disorder (MDD) is a prevalent and debilitating mental illness with a substantial health and economic burden (Lam et al., 2016). Approximately 280 million people in the world, or 5% of all adults, suffer from MDD (Institute of Health Metrics and Evaluation, 2019). Its clinical manifestations are characterized by major depressive episodes (MDEs) – periods of persistently low mood and anhedonia typically lasting for at least 2 weeks at a time. More than 30% of individuals with MDD receiving adequate dose and duration of pharmacotherapy become treatment-resistant, and of those who do respond to treatment, over 65% never reach remission (Rush et al., 2006). Similarly, only 25% of treatment-resistant individuals receiving intensive psychotherapy reach remission (Schatzberg et al., 2005), and capacities in such programs are often insufficient to meet the demand. The magnitude of the MDD burden largely reflects the limited effectiveness of available treatment. Given the critical nature of the illness, with cases often involving suicidal ideation and a lack of an optimal therapeutic outcome (i.e., remission), there is an urgent need for more effective, scalable treatment strategies.

### 1.1 Brain stimulation: the need for optimal targeting

Antidepressants are considered to be the first-line treatment for moderate-to-severe MDD (Anderson et al., 2008). However, many individuals do not respond to these medications and meet the criteria for treatment-resistant depression (TRD); thus, alternative therapeutic options have been explored. In particular, brain stimulation techniques have been used in cases where first-line therapy has not been effective or feasible. One well-established, effective non-invasive brain stimulation (NIBS) technique that is approved by the United States Food and Drug Administration (FDA) for the treatment of severe depression is electroconvulsive therapy (ECT). However, this technique requires anesthesia and seizure induction. As such, some individuals may be apprehensive about undergoing ECT due to its potential side effects, such as memory loss, as well as the associated stigma and a lack of understanding of the ECT procedure itself. Deep brain stimulation (DBS), which is FDA-approved for movement disorders such as Parkinson’s disease and essential tremor, as well as dystonia, epilepsy, and obsessive-compulsive disorder, has also shown promise in treating depression, specifically TRD, although clinical trials have produced mixed results (Hitti et al., 2020; Figee et al., 2022). Irrespective of its potential efficacy, DBS is an invasive procedure that can lead to surgical complications and adverse effects, making it suboptimal to a substantial number of treatment candidates (Boviatsis et al., 2010; Fenoy and Simpson, 2014; Moro, 2016). Furthermore, the administration of DBS requires personnel with a high degree of specialization, which limits its accessibility. These factors warrant the investigation of effective brain stimulation techniques that would overcome these limitations.

Recently, several other NIBS techniques have received increased attention in depression research, as they may overcome the aforementioned limitations of ECT and DBS while achieving comparable therapeutic effects. Some notable examples include transcranial electrical stimulation (TES) and transcranial magnetic stimulation (TMS), the latter of which is FDA-approved for the treatment of TRD. Through the modulation of neuroplasticity, these NIBS modalities induce long-lasting changes in the excitability of brain regions involved in regulating thoughts, emotions, and behavior (Boes et al., 2018; Polanía et al., 2018). The therapeutic potential of NIBS stems from its ability to evoke immediate and sustained directional modulation of neural network activity in either an excitatory or inhibitory fashion (Miniussi et al., 2013; Cantone et al., 2017) or by modifying the threshold of synaptic plasticity (Karabanov et al., 2015; Hassanzahraee et al., 2018). Several meta-analyses and systematic reviews have demonstrated the efficacy and tolerability of both TES and TMS for the treatment of depression (Berlim et al., 2014; Kedzior et al., 2014; Dunlop et al., 2017; Pacheco et al., 2021). Furthermore, response and remission rates for these modalities remain in the range of 33%–45% and 16%–22%, respectively (Cao et al., 2018; Razza et al., 2020), although individual studies have shown much higher remission rates, such as with transcranial alternating current stimulation (tACS) in MDD (54% remission) (Wang et al., 2022) or the Stanford Accelerated Intelligent Neuromodulation Therapy (SAINT) protocol for neuronavigated intermittent theta-burst repetitive TMS in TRD (57%–86% remission) (Cole et al., 2020, 2022).

Safety and tolerability are considered to be relative advantages of NIBS, as risks and adverse events have been well studied over the past 25 years and are reflected in international consensus safety guidelines (Nitsche et al., 2003; Poreisz et al., 2007; Rossi et al., 2009). However, when deployed with the aim of inducing excitability changes in deeper brain structures, such as the limbic areas or basal ganglia, most NIBS techniques, including TES and TMS, are unable to reach these structures without inadvertently co-stimulating more superficial brain tissue. Due to the physical properties of applied electric current or magnetic field, focality decreases with increasing depth, and neuromodulation of deeper brain tissue is achieved at the expense of applying stronger stimulation of the overlying cortex, which, aside from non-specific targeting alone, may lead to adverse effects and a breach of safety guidelines (Deng et al., 2013). Due to this depth/focality trade-off, TMS and TES are limited to the stimulation of relatively superficial cortical regions.

Given these issues, much excitement has surrounded a major new technological development in the world of NIBS – second-generation brain stimulation techniques that rely on acoustic, optical, magnetic, and electric signals (Liu et al., 2022). These include focused ultrasound, near-infrared optogenetic, and nanomaterial-enabled magnetic stimulation, all of which offer great prospects for neuromodulation. Another technique is transcranial temporal interference stimulation (tTIS) – a form of tACS that uses combinations of spatially interacting electric fields to target deep brain structures with markedly reduced stimulation of the overlying cortex (Grossman et al., 2017, 2018). The translational potential of tTIS to provide improved treatment for neurological and psychiatric disorders follows its purported neuromodulatory mechanisms that are similar to other brain stimulation techniques but with two distinct advantages – non-invasiveness, as opposed to DBS, and spatial accuracy, as opposed to TES and TMS (Lozano, 2017; Grossman et al., 2018).

### 1.2 Transcranial temporal interference stimulation

While the literature on tACS in depression research is nascent in comparison to TMS, tACS serves as a promising NIBS technique with high therapeutic potential and minimal side effects, with clinical trials for MDD already emerging (Alexander et al., 2019; Wilkening et al., 2019; Haller et al., 2020; Riddle et al., 2022). However, the electric fields induced by conventional tACS are fairly broad (Datta et al., 2009; Rampersad et al., 2014), making it difficult to target specific brain regions with high focality. Moreover, electric fields applied with tACS dissipate with the traveled distance, so the stimulation of desired deep brain structures unavoidably results in unwarranted co-stimulation of the overlying cortex.

When applied at a frequency that corresponds to the frequency range of endogenous neural oscillations (0.1–80 Hz) and in the so-called “ripple range” (140 Hz) (Moliadze et al., 2010), tACS may be able to interact with ongoing rhythms in the cortex by synchronizing them with the applied tACS current sine wave, which itself acts as an exogenous oscillation. When applied in the low kHz range (1–5 kHz), tACS is able to selectively target the membrane excitability of neurons by altering the accumulation of calcium in the presynaptic nerve terminals (Citri and Malenka, 2008), but this kHz range does not entrain with or synchronize neuronal oscillatory activity due to the low-pass filtering properties of the neuronal cell membrane (Antal and Paulus, 2013). On the other hand, tTIS involves the simultaneous application of two or more independent sinusoidally oscillating current waveforms at slightly different high frequencies, which alone do not influence neuronal activity due to the neuronal membrane’s low-pass filter (Grossman et al., 2017, 2018; Lozano, 2017). The combination of two high-frequency sine waves of slightly different frequencies (e.g., 1,000 and 1,130 Hz), however, generates two electric fields that overlap and form a high-frequency carrier wave (the average of the two input frequencies, 1,065 Hz) that is modulated by a low-frequency envelope oscillating at the much lower “beat” frequency that represents the absolute difference of the two sinusoids (e.g., 130 Hz). tTIS effects depend on this “beat” interaction to drive neuromodulation. Since the strength of the stimulation at any given location is determined by the electric field that is lower in amplitude, the resulting focus is much smaller and enables better targeting. Mechanistically and much like for conventional tACS, it has been shown that tTIS alters the timing, but not the rate, of neuronal spiking activity, with the exception that tTIS offers a way to disrupt pathological oscillatory activity focally and reduce neural synchrony in deep brain structures (Vieira et al., 2024).

This concept was formed and validated by Grossman et al. (2017) using rodent models (Grossman et al., 2017). They performed two experiments with the aim of stimulating the mouse hippocampus through the surface of the skull. The first experiment delivered direct 10 Hz stimulation, and the second experiment delivered 2,000 and 2,010 Hz currents to achieve a “beat” frequency of 10 Hz. With the 10 Hz current, both the hippocampus and cortex were engaged, as indicated by the presence of *c-fos* expression, whereas with the tTIS “beat” frequency of 10 Hz, only the hippocampus was activated. Furthermore, finite element modeling of simulations of tTIS electric fields in human anatomical models suggests that large deep brain structures, such as the hippocampus or subgenual anterior cingulate cortex (sgACC), could also be selectively targeted (Grossman et al., 2017, 2018; Rampersad et al., 2019).

For the past 70 years, temporal interference stimulation (TIS) has been employed in research, typically under the name of *interferential current therapy*, as an effective supplemental tool for physical rehabilitation by electrically stimulating deep muscle tissue (Goats, 1990; Fuentes et al., 2010). More recently, TIS has been used as a neurorehabilitation tool, particularly in the treatment of Parkinson’s disease, Alzheimer’s disease, stroke, and aphasia (Brown, 1975; Goats, 1990; Fuentes et al., 2010; Moore et al., 2018; Skipper et al., 2018; Hussein et al., 2022). The safety profile of TIS has been demonstrated in several preclinical studies, indicating that it does not induce DNA damage, changes in synaptic density, or changes in tissue temperature, nor does it alter the intensity and density of immunohistochemically stained neurons (Grossman et al., 2017; Esmaeilpour et al., 2021; Missey et al., 2021; Sunshine et al., 2021). Modifications to the physics of TIS have also been actively explored. For instance, square electric fields creating a pulse-width modulated interfering electric field have been shown to stimulate neuronal activity as effectively as conventional tTIS with sinusoidal electric fields (Luff et al., 2024). Moreover, the introduction of a phase-canceling electric field appears to improve the focality of tTIS and reduce off-target stimulation (Savvateev et al., 2023).

Several studies have also shown the successful use of tTIS in humans. Devaney et al. (2020) were the first to demonstrate tTIS safety in the human visual cortex using a “beat” frequency of 10 Hz, which was well tolerated in the entire sample and resulted in no disturbances in visual perception (Devaney et al., 2020). Several studies followed suit and demonstrated the feasibility, safety, and functional engagement of stimulated targets with tTIS in healthy humans (Wessel et al., 2021; Ma et al., 2022; Piao et al., 2022; von Conta et al., 2022; Zhang et al., 2022; Zhu et al., 2022); most of these studies, however, aimed to engage superficial primary cortices as targets where other NIBS modalities, such as TES or TMS, are already capable of inducing neuromodulation. Three more recent studies successfully showed the engagement of deep brain structures in healthy humans with tTIS, notably the hippocampus (Violante et al., 2023) and striatum (Wessel et al., 2023; Vassiliadis et al., 2024a). In these studies, target engagement was confirmed through independent evaluation of task-based functional magnetic resonance imaging (fMRI) and a behavioral experiment, and these results lay the groundwork for designing a subsequent round of human tTIS studies with alternative targets. Notably, the work by Vassiliadis et al. (2024a) showed that tTIS was able to selectively modulate striatal mechanisms involved in motor reinforcement learning in humans, using tTIS as a tool to highlight the causal relationship between the function of this deep brain structure and human behavior. In regard to the feasibility of deep tTIS in humans, findings from 119 human participants and >250 stimulation sessions support its safety and tolerability profile and ascertain its excellent blinding efficiency for sham-controlled designs (Vassiliadis et al., 2024b).

Taken together, tTIS offers a novel opportunity over tACS in terms of targeting accuracy and specificity. First, because the tTIS field at its beat frequency, which is considered the component that may result in neuromodulation, can reach deep brain structures with higher field strengths than in the overlying areas. Second, because this field has a smaller focus than regular tACS for deep as well as superficial brain regions.

### 1.3 Therapeutic target: subgenual anterior cingulate cortex

As the first clinical trial to evaluate the prospects of tTIS for MDD, we selected the sgACC [Brodmann Area 25 (BA25)] as our target of interest based on its likely role in MDD symptomatology and treatment response, as well as its rich connectivity profile (Mayberg, 2009). Antidepressant response to active and placebo pharmacotherapy, cognitive-behavioral therapy (CBT), and ECT manifests itself in target engagement of the sgACC (Mayberg et al., 1999; Chen et al., 2007; Mayberg, 2009), and functional hyperactivity of this region best characterizes more treatment-resistant patients (Seminowicz et al., 2004; Mayberg et al., 2005; Greicius et al., 2007). Importantly, high-frequency (>100 Hz) DBS of the sgACC, which mimics the stimulation effects of tTIS proposed in this clinical trial protocol, leads to a long-term response and remission in TRD cohorts (Holtzheimer et al., 2017; Sobstyl et al., 2022). Depressed patient samples are characterized by anatomical sgACC changes on structural MRI scans as well as post-mortem identified glial cell abnormalities (Drevets et al., 1997; Öngür et al., 1998). Moreover, structural and functional variability in this region has been linked to a normal polymorphism in the serotonin transporter, an emerging risk factor for depression (Pezawas et al., 2005). Overall, converging anatomical and histological findings complement broad functional imaging literature, which links sgACC activity to the regulation of negative emotional states. This has been illustrated by sgACC hyperactivity observed in response to provoking sad mood through tryptophan depletion (Ghashghaei et al., 2007), passive exposure to emotionally charged stimuli (Zald et al., 2002; Siegle et al., 2006), and autobiographical memories (Mayberg et al., 1999).

The basis of a more specific role of the sgACC in MDD is grounded in abundant afferent and efferent connections between the sgACC and the insula, brainstem, and hypothalamus, evident in autonomic and circadian components of emotion regulation, including stress response, as well as alterations in sexual functioning, sleep, appetite, neuroendocrine, and neuroimmune functioning (Freedman et al., 2000; Vertes, 2004; Ghashghaei et al., 2007; Hsu and Price, 2007). Reciprocal pathways linking the sgACC BA25 to orbitofrontal, medial frontal, and dorsal prefrontal cortices, the anterior and posterior cingulate cortices, and the amygdala, hippocampus, and nucleus accumbens further identify plausible pathways through which interceptive and homeostatic processes might influence aspects of learning, memory, reward, and reinforcement (Haber et al., 2006; Petrides and Pandya, 2007), which are the core cognitive and behavioral components impaired in depressed individuals. These structural and functional connections display considerable overlap with patterns of regional changes observed in response to pharmacotherapy and CBT, providing a solid foundation to pursue strategies that might effectively alter the sgACC connectivity in individuals with MDD rather than focusing on merely normalizing the absolute activity of the sgACC in isolation. The repeated observations of cellular abnormalities in the sgACC of depressed patients post-mortem, studies of acute emotional states, and predictable decrease in the sgACC activity in response to pharmacological and somatic antidepressant treatments provide a solid foundation to test the therapeutic potential of direct modulation of the sgACC (BA25) using high-frequency tTIS as a novel intervention strategy.

## 2 Objectives and hypotheses

The overarching goal of this study is to continue establishing the prospects of tTIS in humans and provide novel evidence for the impact of tTIS on metrics of the sgACC target engagement across several neuroimaging modalities. This study also intends to establish the feasibility, tolerability, and therapeutic potential of repetitive tTIS for MDD, thus pioneering the investigation of a course of tTIS sessions in a clinical population. Furthermore, it will attempt to establish a causal relationship between the manipulation of the sgACC with tTIS and changes in its target engagement metrics during the resting state, as well as electrophysiological and clinical correlates of depressive symptoms.

Our primary aim is to demonstrate target engagement of the sgACC with tTIS in participants with MDD using the resting-state fMRI, arterial spin labeling (ASL), and diffusion-weighted MRI, as evidenced by pre- vs. post-treatment changes in its intrinsic activity, functional connectivity (FC), perfusion, and anatomical connectivity with other brain regions. We hypothesize that in participants with MDD, a course of 10-day active 130 Hz tTIS will induce engagement of the sgACC post-treatment, which will be superior to sham. Target engagement will be measured as pre- vs. post-treatment change in: (a) resting-state blood-oxygen-level-dependant (BOLD) activity, quantified by power in the BOLD characteristic frequency range (0.015–0.08 Hz), (b) perfusion metrics of cerebral blood flow (CBF), and (c) BOLD activity and CBF in resting-state functional and anatomical networks, or brain regions that are strongly coherent with the sgACC functionally and connected anatomically. We hypothesize that we will observe (a) an increase in BOLD activity in sgACC voxels in tTIS arms but not the sham due to the direct stimulation of the area, (b) an increase in CBF in sgACC voxels in tTIS arm but not the sham due to the direction stimulation of the area, (c) an increase in FC between sgACC and dorsolateral prefrontal cortex (dlPFC), ventromedial prefrontal cortex (vmPFC), and insula – the nodes that display reduced baseline resting-state FC in unmedicated MDD patients compared to healthy controls (Murrough et al., 2016; Wang et al., 2018; Cheng et al., 2021), (d) a significant association between changes in the FC of the sgACC with the abovementioned nodes and anatomical connectivity. Our secondary aim is to demonstrate, using a randomized sham-controlled design, the feasibility, tolerability, and therapeutic potential of repetitive tTIS in participants with MDD. We hypothesize that a 10-day active 130 Hz tTIS will produce significantly greater improvement than sham stimulation in scores of standardized clinical measures of mood symptoms severity in MDD, anxiety, quality of life, and functional impairment. We will also perform exploratory analyses of (a) pre- vs. post-stimulation changes in whole-brain resting-state FC of the sgACC, (b) pre- vs. post-stimulation changes in resting-state electroencephalography (EEG) spectral power after a single session of tTIS and over the course of 10 sessions, (c) pre- vs. post-stimulation changes in the amplitude and latency of the auditory mismatch negativity (MMN) event-related potentials (N1 and P1) after a single session of tTIS and over the course of 10 sessions, (d) feasibility of performing concurrent EEG acquisition during tTIS (termed tTIS-EEG), and (e) source signal propagation during concurrent tTIS-EEG using our proposed configuration for the stimulation of the sgACC with tTIS.

## 3 Methods and analysis

### 3.1 Ethics oversight

This study has been approved by the UHT Research Ethics Board (Approval No.: 21-152) and will be conducted in compliance with the principles of the Tri-Council Policy Statement: Ethical Conduct for Research Involving Humans (TCPS 2) (Canadian Institutes of Health Research, Natural Sciences and Engineering Research Council of Canada, and Social Sciences and Humanities Research Council, 2018) and International Council for Harmonization Good Clinical Practice E6 (ICH GCP E6) (Vijayananthan and Nawawi, 2008). The trial has been registered on ClinicalTrials.gov (NCT05295888).

### 3.2 Participant selection and recruitment

The trial will enroll individuals with moderate-to-severe MDD in a MDE. Treatment resistance is not a criterion for inclusion. Participants, if receiving any treatment, must remain on a stable dosage of any concomitant medications and shall have no changes to their treatment regimen 4 weeks before enrollment and during the trial. The protocol prohibits certain medications that may interfere with the effect of NIBS therapies, such as barbiturates, benzodiazepines, and certain anticonvulsants (Kaster et al., 2020). The key eligibility criteria are displayed in Table 1, and the full list is available in Supplementary Table S1.

**TABLE 1:** Summary of eligibility criteria for the clinical trial examining transcranial temporal interference stimulation in major depressive disorder.

**Inclusion criteria**
(1) Male or female, 18–65 years of age (inclusive)
(2) Meet the DSM-5 criteria for MDD with a current MDE without psychotic features, as confirmed by the MINI
(3) MADRS total score of ≥20 (moderate-to-severe MDD)
(4) No change in treatment regimen in the 4 weeks prior to screening
**Exclusion criteria**
(1) Any psychiatric disorder, including substance use disorder, other than MDD or comorbid anxiety disorder due to its high co-occurrence with MDD
(2) Any neurological disorder, serious medical illness, sensory abnormality, or unstable clinical finding
(3) Active suicidal intent, confirmed by the MINI Module B (Suicidality) or MADRS item #10 score ≥4
(4) Take medications prohibited by the protocol that may interfere with the effect of tTIS
(5) Pregnant or lactating
(6) An intracranial implant (e.g., aneurysm clips, shunts, stimulators, cochlear implants, or electrodes) or any other metal object within or near the head, excluding the mouth, that cannot be safely removed
(7) Change in medication or psychotherapy treatment regimen before screening
(8) Contraindications for receiving tTIS or undergoing a magnetic resonance imaging scan

DSM 5, Diagnostic and Statistical Manual of Mental Disorders, 5th edition; MADRS, Montgomery-Åsberg Depression Rating Scale; MDD, major depressive disorder; MDE, major depressive episode; MINI, Mini International Neuropsychiatric Interview; tTIS, transcranial temporal interference stimulation.

### 3.3 Study design

**FIGURE 1:**
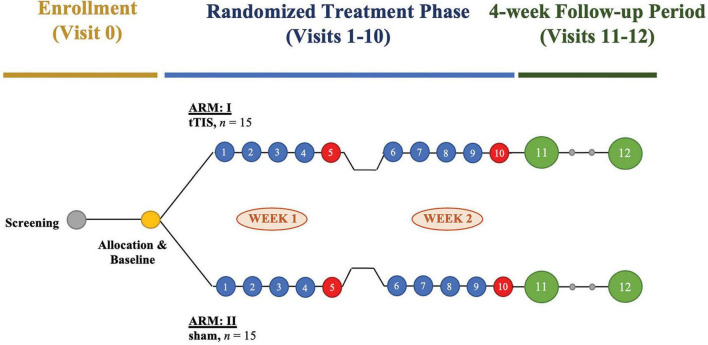
Study design of a pilot randomized sham-controlled interventional clinical trial examining the target engagement and therapeutic potential of transcranial temporal interference stimulation (tTIS) in major depressive disorder (MDD). The trial will enroll 30 participants with MDD, who will be randomized to receive active tTIS or sham stimulation in 1:1 allocation. Participants will complete 10 sessions of tTIS or sham stimulation over 2 weeks. A comprehensive clinical assessment with a semi-structured interview will take place on treatment days 5 and 10 after the stimulation (red circles). After randomized treatment, participants will undergo a post-stimulation visit and a delayed follow-up visit (green circles) scheduled 5 and 20 days after the last treatment day 10, respectively.

This study is a pilot single-site randomized sham-controlled interventional clinical trial based at the Interventional Psychiatry Program, St. Michael’s Hospital – Unity Health Toronto (UHT) in Toronto, ON, Canada. The trial will compare a course of 10 sessions of 130 Hz tTIS stimulation vs. 10 sessions of sham stimulation, delivered over 2 weeks (Figure 1). Thirty participants with MDD will be recruited into the trial and randomized into two parallel arms (active tTIS vs. sham) in a 1:1 fashion. Block randomization will be stratified by sex to allocate participants using a secure randomization module on Research Electronic Data Capture (REDCap) (Harris et al., 2009), as sex differences in brain volume and skull anatomy influence NIBS treatment outcomes (Hanlon and McCalley, 2022). Participants, investigators, outcome assessors, and data analysis will be blinded to treatment allocation. A randomization table will be generated using an external tool and uploaded to REDCap. Once a new participant is ready to be randomized, a research coordinator external to the study will enter their sex (a stratification variable) into REDCap, and the system will use the pre-defined randomization table to assign them to a treatment group.

### 3.4 Sample size considerations

Based on studies in TES with fMRI, block design, and use of computational iterative neurostimulation (Ghobadi-Azbari et al., 2021), we expect that 30 participants will have adequate power to demonstrate target engagement of the sgACC. The proposed sample size (*n* = 30) is also based on the overall goal of (a) reasonably assessing the feasibility outcomes and (b) estimating the variability of the clinical outcome measures and is consistent with published recommendations for pilot trials designed to estimate standard deviations for larger future randomized controlled trials when the effect size is small-to-medium (Lancaster et al., 2004; Whitehead et al., 2016). As such, based on sample sizes used in pivotal TES studies (Berlim et al., 2013), we expect that 30 participants will be adequate to demonstrate the feasibility, tolerability, safety, and therapeutic effect of tTIS. Moreover, the pilot study will provide effect size estimates to design a definitive efficacy trial.

For this pilot study, to have a 90% power (i.e., being 90% confident that the effect is true and that it is not a type I/II error), the top end of the two-sided 95% confidence intervals will be the maximum confidence of having a true difference between active tTIS and sham arms. If there is no difference between active tTIS and sham arms, then *p* ≥ 0.051 will indicate no difference with a 90% confidence, and if there is a difference, then *p* ≤ 0.05 will indicate the difference with a 90% confidence. To have the medium effect size of *f* = 0.25 for the mixed-model analysis of variance (Cohen, 2013), 30 patients (15 per group) are required to have a 90% power. We anticipate having a 15% dropout rate in the randomized phase of the study due to side effects or lack of adherence to protocol and approximately a 50% response rate in the acute phase of treatment. A sample size of 15 per group will give us precise estimates of variability in the clinical outcome, namely a change in the Hamilton Depression Rating Scale 17-Item (HAM-D-17) (Hamilton, 1960) score over time (Korn et al., 2012; Cohen, 2013), and this sample size is within the recommended total sample sizes for a precise estimate of the standard deviation and correlation for HAM-D-17 scores to use in the future sample size calculation for a definitive larger scale trial (between *n* = 24 and 50) (Julious, 2005; Sim and Lewis, 2012; Billingham et al., 2013).

### 3.5 Data and safety monitoring

Adverse Event (AE) data will be collected using a standard form at each visit. Additionally, a standard questionnaire for tTIS-related AEs will be completed before and immediately after each treatment session. If a participant indicates to experience intolerable side effects during stimulation, stimulation will be terminated immediately. If severe side effects are reported during or after stimulation in 2 consecutive sessions, or in more than 2 sessions, participation will be terminated. A Data and Safety Monitoring Board will be assembled to monitor participant safety and changes in clinical scores. This panel comprises two independent, external experts. REDCap software (Harris et al., 2009) will be used for data collection and management. Access will be secured by an encrypted virtual private network and protected by multiple levels of authentication. All other identifiable participant data will be stored on a secured internal server.

### 3.6 tTIS stimulation paradigm

The tTIS intervention will be delivered using the High-Definition (HD) Interferential Neuromodulation System (IFS) manufactured by Soterix Medical, Inc. (Woodbridge, NJ, United States). The device is designed to deliver two independent alternating currents to the brain at different frequencies. tTIS will be delivered through two pairs of sintered ring HD electrodes (circular external diameter 1.2 cm) placed on the scalp via a 10-10 HD cap and an HD-GEL (Soterix Medical, Inc.), with each electrode pair delivering continuous high-frequency sinusoidal waveforms at frequencies of 1,000 and 1,130 Hz, respectively, resulting in a carrier frequency of 1,065 Hz and a “beat” frequency of 130 Hz. This stimulation paradigm was designed to mimic the frequency used in DBS of the sgACC for TRD (Mayberg et al., 2005; Khairuddin et al., 2020). The current amplitude will be 2 mA per electrode pair, i.e., the injected current will sinusoidally vary between 0 and 2 mA. The stimulation will be delivered for 30 min, which includes 30-s ramp-up and ramp-down periods at the start and end. For sham stimulation, the current will be ramped down to 0 mA directly after ramping up, mimicking the sensations associated with verum stimulation. Based on our prior experience, we expect to see impedances between 1 and 10 kΩ during stimulation, which is tolerated well by participants. If the impedance of any stimulation channel exceeds 25 kΩ, the stimulation device will give a warning, upon which the experimenter will add gel to reduce the impedance (without stopping the stimulation). If any impedance exceeds 50 kΩ, the channel quality indicator will display red, indicating that the impedance is outside of the preferred range. At this time, stimulation will not cease, and the reduced output current will be delivered in relation to the maximum compliance voltage of the stimulator.

### 3.7 Modeling

**FIGURE 2:**
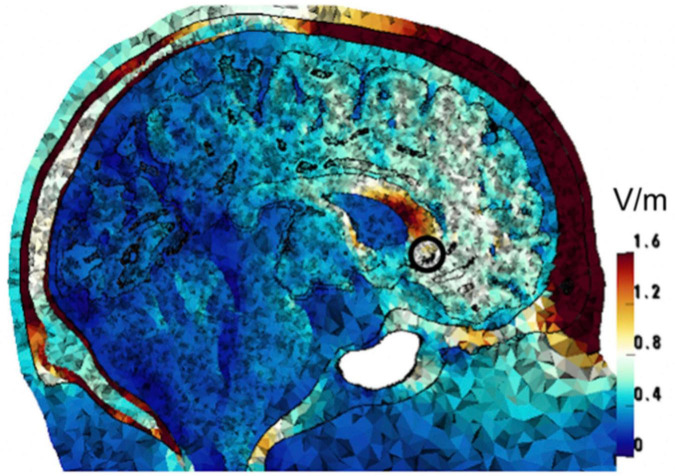
Computational optimization of tTIS electrode placement. The image shows one of three head models for which the tTIS field strength and focality were optimized. The color scale represents the optimized tTIS field strength distribution in the head. The target region (sgACC) is marked with a black circle.

Computational modeling was performed to find the 4-electrode tTIS configuration that achieves the highest field strengths in the sgACC. Targeting of the sgACC, therefore, is assumed based on finite element simulations, and has not been previously verified experimentally. Following standard methods, volume conduction models were constructed from MRI images of three healthy adult males, and simulations were performed to calculate the electric fields induced in the head models by injecting current through electrodes on the scalp (Rampersad et al., 2019). Such simulations were performed for 146 million different current patterns, each consisting of 4 electrode locations and current amplitude values for each electrode. The stimulation target was defined as the confluence of three white matter tracts (uncinate fasciculus, cingulum, and forceps minor), corresponding to the location of the sgACC in each hemisphere. This area was marked in the images on which the models were based and then integrated into the models. The current pattern that achieved the highest field strength at the “beat” frequency in this target region was then selected for each model, with a Pareto optimization step to reduce fields in off-target areas, *given the primary objective to maximize field strength in the target*. It is not possible to reduce off-target fields further without reducing the target fields (Rampersad et al., 2019). We used the intersection of the optimal configurations of three models to create a symmetric set of four electrodes. This resulted in electrodes placed at the AF7, T7, AF8, and T8 locations of the international 10-10 system. Figure 2 shows the tTIS electric field strength on a sagittal cut through the target region in one of the head models. With 2 mA (peak) current supplied through each electrode pair, the resulting tTIS electric field strength in the target area was 0.60 V/m. Based on prior modeling and experimental studies of tACS (Rampersad et al., 2019), this should be sufficient to obtain neuromodulatory effects. Some areas of the brain receive higher field strengths than the target region. Due to the geometry of the brain and highly conductive cerebrospinal fluid, this cannot be avoided (Rampersad et al., 2019). However, the current paradigm is optimal for the purpose of this clinical trial: within the set of solutions examined, no other current pattern produced higher tTIS field strength in the sgACC than the one proposed. It should be noted that conventional stimulation, such as tACS, would produce much larger off-target fields even with optimization (see Figures 5 and 11 in Rampersad et al., 2019).

### 3.8 Study procedures

Participants will complete telephone pre-screening and, if eligible, will be invited to the hospital to undergo urine tests for the presence of pregnancy and substance use, which will be screened out. The screening will involve the review of eligibility criteria, demographics information, medical history, psychiatric history, treatment history, confirmation of MDD diagnosis and the presence of moderate-to-severe symptoms, and a review of safety considerations to receive rTIS or undergo an MRI. If all eligibility criteria are met, written informed consent will be obtained, and participants will be randomized into one of the treatment arms (active tTIS vs. sham). All participants will have 13 on-site visits: screening (Visit 0), baseline combined with day 1 of tTIS (Visit 1), days 2–10 of tTIS (Visits 2–10), and two separate follow-up visits after the last day of tTIS during weeks 3 (Visit 11) and 6 (Visit 12). Visits will be completed on weekdays (Monday to Friday) for compliance and convenience. At baseline (Visit 1) and the last day of tTIS (Visit 10), participants will undergo a 60-min MRI session for the assessment of sgACC target engagement; this session includes the acquisition of anatomical, resting-state functional, perfusion, and diffusion scans. At Visits 1, 5, and 10, resting-state and task-based EEG data will be collected for assessment of changes in brain oscillations that tTIS is likely to modulate (Esmaeilpour et al., 2021). Most clinical assessments will be completed at Visits 5 and 10 to reduce participant burden. The primary outcome time point will be Visit 10, corresponding to the time point at which post-treatment MRI and clinical assessments will be completed. Refer to Table 2 for the study schedule, clinical assessments, and study procedures conducted during each visit.

**TABLE 2:** Proposed schedule of assessments and procedures.

	Study period													
	Enrollment	Baseline	Randomized treatment phase										Follow-up period	
			Week 1					Week 2					Week 3	Week 6
Visit	0	1	1	2	3	4	5	6	7	8	9	10	11	12
**Screening**														
Eligibility	X													
Informed consent	X													
DEMO	X													
MINI	X													
ATHF	X													
MADRS	X													
tTIS/MRI safety	X	X										X		
Urine test	X													
EHI	X													
Allocation		X												
**Interventions**														
A – tTIS (30 min)			X	X	X	X	X	X	X	X	X	X		
B – Sham (30 min)			X	X	X	X	X	X	X	X	X	X		
**Assessments**														
HAM-D-17		X					X					X	X	X
QIDS-SR-16		X	X	X	X	X	X	X	X	X	X	X	X	X
AE			X	X	X	X	X	X	X	X	X	X	X	X
GAD-7		X					X					X	X	X
WHO-5		X					X					X	X	X
SDS		X					X					X	X	X
CONMED		X					X					X	X	X
tTIS-E		X					X					X	X	X
BOAGF							X					X	X	X
**Procedures**														
MRI		X										X		
EEG			X				X					X		

AE, Adverse Events Log; ATHF, Antidepressant Treatment History Form; BOAGF, Blinding Outcome Assessor Guess Form; CONMED, concomitant medications record; DEMO, demographics; EEG, electroencephalography; EHI, Edinburgh Handedness Inventory; GAD-7, Generalized Anxiety Disorder 7-Item; HAM-D-17, Hamilton Depression Rating Scale 17-Item; MADRS, Montgomery-Åsberg Depression Rating Scale; MINI, Mini International Neuropsychiatric Interview; MRI, magnetic resonance imaging; tTIS, transcranial temporal interference stimulation; tTIS-E, Transcranial Temporal Interference Stimulation Expectancy and Experience Questionnaire; QIDS-SR-16, 16-Item Quick Inventory of Depressive Symptomatology – Self-Report; SDS, Sheehan Disability Scale; WHO-5, World Health Organization-Five Well-Being Index.

### 3.9 Clinical assessments

An independently trained rater will administer psychometric instruments, which will be used to assess the effect of tTIS on MDD clinical outcomes. Symptoms of depression will be assessed using the HAM-D-17 (Hamilton, 1960) and 16-Item Quick Inventory of Depressive Symptomatology – Self-Report (Rush et al., 2003). Other measures include validated scales of anxiety [Generalized Anxiety Disorder 7-Item (GAD-7)] (Spitzer et al., 2006), quality of life [World Health Organization-Five Well-Being Index (WHO-5)] (Bech et al., 1996, 2003), and functional impairment [Sheehan Disability Scale (SDS)] (Sheehan et al., 1996). Refer to Table 2 for the frequency of administration. Participants’ expectancy and experience with tTIS and the effectiveness of the rater’s blinding will also be evaluated.

### 3.10 Magnetic resonance imaging

At Visits 1 and 10 (baseline vs. post-treatment), participants will undergo a 60-min MRI (Siemens 3.0 T Skyra) session with a structural and functional protocol using a 32-channel head coil. The MRI protocol comprises: (1) a high-resolution T1-weighted anatomical scan acquired using a magnetization-prepared rapid gradient-echo (MP-RAGE) sequence; (2) a series of B_0_ field map scans to correct the images for signal distortion; (3) fMRI images using a whole-brain T2*-weighted BOLD echo-planar imaging series during the awake resting-state in the eyes-open condition and while viewing a fixation cross (Raichle and Snyder, 2007); (4) a whole-brain diffusion-tensor imaging series; and (5) a 3D pseudo-continuous arterial spin labeling (pCASL) scan. Cardiovascular and respiratory data will be monitored throughout the entire session and used to correct for MRI signal distortions.

### 3.11 Electroencephalography

Before, during, and after tTIS session at Visits 1, 5, and 10, participants will undergo a 42-min EEG data collection, which includes: (1) pre-stimulation EEG at rest with eyes open (3 min); (2) pre-treatment EEG during the auditory MMN task (Näätänen et al., 1978) (3 min); (3) concurrent EEG acquisition during tTIS (30 min); (4) post-stimulation EEG at rest with eyes open (3 min); and (5) post-stimulation EEG during the auditory MMN task (3 min). A 32-channel BioSemi ActiveTwo EEG acquisition system (Cortech Solutions Inc., Wilmington, NC, United States) will be used to collect surface EEG measurements. The desired sampling frequency is 4,096 Hz. Each sample will be quantified at 16 bits, and the digital data will be stored in a secured server for further signal analysis of time and spectral domain features. Auditory MMN is an event-related potential that appears in neurophysiological recordings during a sequence of repetitive sounds that is interrupted by an occasional “oddball” sound, which differs from other sounds in duration or frequency (Garrido et al., 2009). The MMN reflects the flow of the current through NMDA receptor-mediated ion channels and could be used as a biomarker to test whether new interventions influence NMDA receptor responsiveness in study participants (Näätänen, 2001; Garrido et al., 2009; Zhou et al., 2013).

### 3.12 Analysis plan

#### 3.12.1 Primary outcomes: target engagement

BOLD activation (power in the 0.015–0.08 Hz frequency range), CBF, seed-based resting-state FC, and anatomical connectivity of the sgACC will be the primary outcome measures to demonstrate sgACC target engagement with a course of tTIS. We hypothesize that we will observe an increase in the BOLD activation and CBF in the sgACC voxels in the tTIS arm but not the sham and an increase in resting-state FC between the sgACC and bilateral dlPFC, vmPFC, and insula in the tTIS arm but not the sham. We also hypothesize that changes in the resting-state FC will be associated with changes in anatomical connectivity between the sgACC and the abovementioned brain regions. Additional exploratory analyses of changes in the whole-brain FC of the sgACC and their association with anatomical connectivity will be performed.

T1-weighted and T2*-weighted images will be preprocessed, with steps including volumetric segmentation and surface extraction, normalization, motion correction, field unwarping, bias field correction, and brain extraction (Esteban et al., 2019). Preprocessing and denoising will be performed using tedana: TE Dependent ANAlysis v.23.0.1 pipeline^1^ (Kundu et al., 2012, 2013; DuPre et al., 2021), which includes slice timing correction, estimation of motion correction, spatial normalization, signal distortion correction, smoothing, and filtering. The left and right sgACC will be identified using the Desikan-Killiany (FreeSurfer) anatomical labeling scheme (Desikan et al., 2006). Pre- vs. post-treatment changes in sgACC time series properties, such as BOLD activity, will be assessed using Wilcoxon signed-rank tests or paired *t*-tests on signal variance at each sgACC voxel. pCASL scans will be used for perfusion detection using the BASIL toolset to conduct simple CBF quantification in the sgACC voxels (Chappell et al., 2009). In addition, seed-based resting-state sgACC FC maps will be computed by calculating Pearson correlation coefficients between the BOLD time series across the rest of the brain, with a focus on bilateral dlPFC, vmPFC, and insula, and the sgACC voxel-averaged seed time series. Using SPM12, 5 mm spherical seeds will be created around the sgACC MNI coordinates, and contrast analysis on the FC will be run using CONN Toolbox v.22a (RRID:SCR_009550, v.22a) (Nieto-Castanon and Whitfield-Gabrieli, 2021). FC-based target engagement will be defined as statistically significant pre- vs. post-treatment changes in these sgACC FC maps, as computed with mass univariate paired *t*-tests.

Regions strongly anatomically connected to sgACC will be identified using diffusion-weighted MRI tractography. For these analyses, raw diffusion-weighted MRI scans will be preprocessed, denoised, and reconstructed using QSIPrep (Cieslak et al., 2021). The preprocessing and denoising steps include conforming image and gradient orientation, head-motion correction, distortion correction, registration, and normalization. White matter tracts can be extracted from the fiber tractography during reconstruction. The cortical terminations of the sgACC-seeded streamlines will define brain regions anatomically connected to the sgACC. We will conduct additional pre- vs. post-stimulation comparisons on those regions specifically in terms of their signal variance and FC patterns, as described above, and with specific interest in the anatomical connectivity between the sgACC and bilateral dlPFC, vmPFC, and insula.

In addition, we will investigate whether these target engagement metrics are associated with clinical outcomes, as quantified by linear regression analyses. Finally, we will compute tTIS electric field models of T1-weighted MRI images to determine *post hoc* the specific pattern of tissue stimulation experienced by each participant. We will test whether these e-field-computed electrical current intensities are correlated with the regional target engagement metrics outlined above using linear regression models.

#### 3.12.2 Secondary outcomes: clinical assessments

Improvement in depressive symptoms will be the main secondary outcome measure. Additional secondary outcome measures will include feasibility, safety, and tolerability metrics of rates of recruitment, withdrawals, and adherence, as well as the number and nature of AEs and serious AEs.

For continuous outcome measures, a mixed-effects model will be used to assess differences in the rate of improvement over time and the final outcome across groups. Treatment group, time, and treatment group × time interaction will be used as explanatory variables, with participant as a random factor. The significance of the treatment group × time interaction effect will be used to test the null hypothesis. For categorical outcome measures, response will be defined as a ≥50% reduction in HAM-D-17 and QIDS-SR-16 total scores at Visit 10. Remission will be defined as a HAM-D-17 total score of <8 at Visit 10. The two-tailed Chi-squared test will be used to assess the significance of any observed differences in the proportion of responders and remitters between groups.

#### 3.12.3 Secondary outcomes: electroencephalography

EEG data will be acquired at Visits 1, 5, and 10 to provide a time-series representation of changes in neural activity over time and after a single session of tTIS. These analyses will be exploratory. Two sets of recordings, before and after the tTIS or sham stimulation, will allow us to examine the immediate effect of the stimulation. Recording EEG signals during the stimulation will offer insights into the feasibility of conducting concurrent tTIS-EEG recordings, as well as source signal propagation.

EEG signal analysis will be done using MATLAB, and the preprocessing steps will include signal noise and artifact removal. Independent component analysis (ICA) will be used to remove electrooculogram (EOG) artifacts. EEG signals will be bandpass filtered at 0.5–40 Hz to remove movement and instrumentation noise. A simple power estimation will be conducted on the resting-state EEG signals. Time and frequency domain analysis will be conducted on segmented auditory MMN EEG signals. The segmentation will divide the EEG signals into short-term epochs of 500 ms (−100 to 400 ms). The analysis will then be conducted on these time segments. Time domain analysis will be used for the auditory MMN tasks to estimate the average difference between deviant and standard ERP responses in order to extract the amplitude and latency of N1 and P1 ERPs. Time domain signal processing techniques will be used to extract statistical metrics such as root mean square, signal variance, and variance of the first derivative of the ERP signals. Frequency domain analysis on the segmented EEG signals will extract energy ratios and power spectral density at each EEG frequency band. Subjective answers obtained as categorical variables will be used in labeling the EEG segments, and machine learning techniques will be implemented to classify the signal segments on the basis of response and remission as well as to determine the combined role of MRI metrics, clinical variables, and EEG signal features in assessing treatment response to tTIS.

### 3.13 Early withdrawal and study termination

Participants are free to withdraw from the study at any time without penalty or loss of benefits. A participant will be discontinued if they miss more than three treatment sessions, are non-compliant, experience worsening depression, meet exclusion criteria at a later time point, experience severe side effects, or if the stimulation device is believed to be unsafe. This study may be terminated if there is sufficient reasonable cause.

## 4 Discussion

The therapeutic effectiveness of NIBS in MDD has been validated across numerous studies, but, with few exceptions (Cole et al., 2020, 2022), its efficacy remains limited due to its dependence on the selection and accuracy of stimulation targets (McLoughlin et al., 2007; Herbsman et al., 2009; Fox et al., 2012; Berlim et al., 2013; Lefaucheur et al., 2017; Liu et al., 2017). Guiding NIBS with cutting-edge imaging techniques is fundamental for locating specific functional brain networks and accurately positioning stimulation sites to fit individual anatomical variations (Mir-Moghtadaei et al., 2015; Luber et al., 2017). To develop NIBS as an individualized treatment for individuals with MDD, determining a combination of suitable imaging methods is crucial for providing optimal antidepressant effects. Our clinical trial is a multi-modal neuroimaging study, where target engagement of the sgACC will be assessed across different modalities and metrics, spanning both structure and function. These data will guide the investigation of the target engagement capabilities of tTIS as a new, emerging technology that can potentially be enabled for personalized and precision psychiatry in the future.

The preliminary studies establishing its safety and effectiveness in healthy humans, as well as its unique spatial accuracy, make tTIS a desirable candidate for NIBS therapy in MDD (Wessel et al., 2021; Ma et al., 2022; Piao et al., 2022; von Conta et al., 2022; Zhang et al., 2022; Zhu et al., 2022). The present study will be the first to demonstrate target engagement of the sgACC and the therapeutic potential of repetitive tTIS in MDD. Data from this trial will provide much-needed safety and effectiveness evidence to the limited literature regarding tTIS for deep brain regions in humans. If successful, our approach of noninvasively targeting sgACC may also be translated to other psychiatric disorders of disrupted brain connectivity characterized by sgACC dysregulation and FC disturbances.

It should be acknowledged, however, that our electrode configuration is based solely on finite element simulations as the configuration that led to the highest tTIS “beat” field strength in the sgACC in three head models. This configuration, ideally, should be validated for target engagement in a single-session experiment among healthy participants using task-based functional neuroimaging. Additionally, off-target high-field strengths are impossible to avoid, given the location of the target and the geometry of the brain. Therefore, the effects of this configuration should also be validated against other brain areas in close proximity to the sgACC, such as the dorsal anterior cingulate cortex. Furthermore, as demonstrated by previous studies that applied tTIS in humans (Violante et al., 2023; Wessel et al., 2023; Vassiliadis et al., 2024a), treatment effects of tTIS can be enhanced if the stimulation is applied during a behavioral task engaging the target region. However, selecting an optimal behavioral task to engage the sgACC remains a challenge, as the seminal literature showing sgACC hyperactivity in depression (George et al., 1995; Mayberg et al., 1999) used transient sadness induction or autobiographical memory tasks, which are highly subjective, difficult to standardize across participants, and lacking quantifiable accuracy responses. Since then, no task has been validated specifically for the engagement of the sgACC, and future work should focus on addressing this shortcoming. In our trial, therefore, changes in absolute BOLD signal may not be as pronounced during the resting-state scan acquisition, although resting-state scans may be more optimal for examining the FC of the sgACC with multiple intrinsic brain networks simultaneously, as well as the association between brain network dynamics and anatomical connectivity (Lv et al., 2018; Al-Arfaj et al., 2023).

The chance to develop new treatments for MDD is an important step in helping people who suffer from this condition. This study may offer individuals with MDD a novel treatment option, which can be beneficial for those who are resistant to antidepressants and psychotherapy or who cannot tolerate other forms of NIBS. Moreover, tTIS is relatively easy to administer: device electronics are simple, and devices can be made smaller for home use. The devices are also low-risk, and patients can be educated on how to self-deliver the stimulation. Unlike its sister technology, TMS, tTIS is a form of tACS that has the potential to be scalable. At-home devices are already emerging, which potentially makes tTIS a cheaper and safer technology. This is advantageous, both in terms of the economics of healthcare provision and the logistics of future large-scale clinical trials. Our study is thus a first step for readapting tTIS for home-based delivery, which will benefit individuals with high functional impairment and limited access to in-hospital treatment programs.

Although participants may experience some degree of relief from mood symptoms due to a course of tTIS, this study has not been designed to benefit the individual participant. Side effects from tTIS/tACS reported in the literature are limited but are not nonexistent; the most common include mild tingling, itching, burning, discomfort or pain, visual sensation, moderate fatigue, skin redness, headache, and difficulties in concentration (Antal et al., 2008; Feurra et al., 2011; Brignani et al., 2013). Due to scheduling restrictions, treatment sessions will only be completed on weekdays, resulting in a 2-day gap between certain sessions. No evidence to date reports this as a limitation, but it is possible that this delay between treatment sessions may affect the treatment outcomes. Furthermore, the stimulation parameters for our tTIS paradigm have been chosen while keeping in mind the goal of minimizing dropout rates in our clinical trial, as tTIS will be administered in a repetitive fashion over 10 days.

The human tTIS studies published to date (Violante et al., 2023; Wessel et al., 2023; Vassiliadis et al., 2024a) are single-session studies in which tTIS was delivered to healthy participants. These studies applied the stimulation at frequencies close to 2 kHz. In our proposed study, tTIS will be delivered at frequencies close to 1 kHz, as it may offer advantages in terms of safety, efficiency, and patient comfort associated with repetitive administration of tTIS – higher frequencies may be associated with more pronounced side effects such as discomfort, phosphenes, and involuntary muscle contractions (Liu et al., 2024). Tissue heating and electrical impedance in biological tissue also increase with frequency (Liu et al., 2024), and using 1 kHz may minimize these risks. In addition, some of the abovementioned studies included tACS as an active high-frequency control instead of the 0-mA sham proposed in this study (Wessel et al., 2023; Vassiliadis et al., 2024a). Absolute stimulation frequencies in the tACS active control arm were equivalent between the two channels and did not generate the neuromodulatory “beat” frequency. This design offers advantages for a basic science experiment aiming to ensure that the target engagement effects are specific to the tTIS “beat” frequency in a desired locus and not due to general electrical stimulation. In our clinical trial, however, the goal is also to ensure any observed change in depressive symptoms is due to the verum tTIS and not other factors such as placebo effects, therapeutic response to high-frequency stimulation, or engagement of off-target superficial cortices with active tACS. Active tACS as a control is likely to lead to a meaningful change in depressive symptoms if areas of the dlPFC are engaged (Alexander et al., 2019; Wilkening et al., 2019; Haller et al., 2020; Riddle et al., 2022), which may mask the true therapeutic effect of active tTIS compared to the absence of active stimulation.

Lastly, although broad network similarities can be seen in MDD, there is considerable person-to-person variance. Future therapeutic approaches should acknowledge and accommodate this fact, including sex-, age-, and head size-specific differences in treatment response. As the tTIS technology develops further, we envisage a more ambitious, effective, personalized therapy design based on individual-level modeling with personalized neuroimaging. Our group-level optimization represents one modest step in this direction, and we hope to perform individual-level tTIS modeling *post hoc* using the acquired MRI scans. If our clinical trial shows successful engagement of the sgACC with tTIS using resting-state MRI, this will warrant further investigation of tTIS effects in MDD using task-based neuroimaging, individual-level modeling, and manipulation of the tTIS parameters space. We aim to encourage the transition in clinical psychiatry away from the trial-and-error, black-box regime of 20th-century medicine and toward personalized medicine, underwritten by computational modeling and precision engineering.

## Footnotes

1
https://zenodo.org/records/7926293
