# Supplementary material for: Target engagement of the subgenual anterior cingulate cortex with transcranial temporal interference stimulation in major depressive disorder: a protocol for a randomized sham-controlled trial
Source: Front Neurosci. 2024 Aug 29;18:1390250. doi: 10.3389/fnins.2024.1390250 (PMC11390435; doi:10.3389/fnins.2024.1390250)
Supplement: Supplementary file 1 [file Data_Sheet_1.DOCX]

Supplementary Material

# Supplementary Tables

**Table S1**. Complete list of eligibility criteria for the clinical trial examining transcranial temporal interference stimulation in major depressive disorder

| **Inclusion criteria** |
| --- |
| (1) provide written informed consent before initiation of any study-related procedures  (2) are outpatients  (3) meet the DSM-5 criteria for major depressive disorder (MDD) with a current major depressive episode (MDE) without psychotic features as confirmed at Screening by the Mini International Neuropsychiatric Interview (MINI)  (4) are male or female, 18 to 65 years of age (inclusive) at screening  (5) have a Montgomery-Åsberg Depression Rating Scale (MADRS) total score of ≥ 20 (moderate to severe depression) at screening.  (6) have had no increase or initiation of any psychotropic medication in the 4 weeks prior to screening  (7) able to adhere to the treatment schedule  (8) pass the tTIS adult safety screening questionnaire  (9) are able to understand and comply with the requirements of the study, as judged by the investigator(s) |
| **Exclusion criteria** |
| (1) have an acute alcohol or substance use disorder, withdrawal symptoms requiring detoxification, or went through detoxification treatment (inpatient or outpatient) within 3 months before Screening, as obtained from MINI, Module I (Alcohol Use Disorder) and Module J (Substance Use Disorder, Non-Alcohol) assessed at Screening  (2) have a concomitant major unstable medical illness, active hepatitis B virus (HBV), hepatitis C virus (HPC), human immunodeficiency virus (HIV), active COVID-19 infection, cardiac pacemaker or implanted medication pump, as per medical history provided by the participant  (3) have active suicidal intent, confirmed by a ‘Yes’ response to Question B3 AND either Question B10 or B11, obtained from the MINI Suicidality, Module B (Suicidality), OR confirmed by the MADRS item #10 score ≥ 4, both assessed at Screening  (4) have a current clinical diagnosis of autism, dementia, or intellectual disability  (5) take medications prohibited by the protocol, such as lorazepam, clonazepam, diazepam, alprazolam, lamotrigine, pregabalin, gabapentin, carbamazepine, topiramate, phenytoin, divalproex / valproate, clobazam, levetiracetam, barbiturates, or other benzodiazepines. The short-acting imidazopyridine zolpidem (5 to 10 mg HS) or the cyclopyrrolone zopiclone (7.5 to 15 mg HS) for insomnia is allowed until midnight on the day before tTIS stimulation  (6) are pregnant or lactating  (7) have any prior or current diagnosis of bipolar I or II disorder, MDD with psychotic features, schizophrenia, schizoaffective disorder, schizophreniform disorder, delusional disorder, or current psychotic symptoms as obtained from MINI, Module C (Manic and Hypomanic Episodes) and Module K (Psychotic Disorders and Mood Disorders with Psychotic Features) assessed at Screening  (8) have any prior or current diagnosis of obsessive-compulsive disorder, post-traumatic stress disorder (current or within the last year), confirmed by MINI and assessed by a study investigator to be primary and causing greater impairment than MDD  (9) have a diagnosis of any personality disorder, and assessed by a study investigator to be primary and causing greater impairment than MDD  (10) have received tTIS for any previous indication due to the potential compromise of subject blinding  (11) have any significant neurological disorder or insult including, but not limited to: any condition likely to be associated with increased intracranial pressure, space-occupying brain lesion, any history of seizure except those therapeutically induced by ECT or a febrile seizure of infancy, cerebral aneurysm, Parkinson’s disease, Huntington’s chorea, multiple sclerosis, significant head trauma with loss of consciousness for greater than 5 minutes, current history of poorly controlled migraines including chronic medication for migraine prevention  (12) have an intracranial implant (e.g., aneurysm clips, shunts, stimulators, cochlear implants, or electrodes) or any other metal object within or near the head, excluding the mouth, that cannot be safely removed  (13) if participating in psychotherapy, have NOT been in stable treatment for at least 3 months prior to entry into the study or anticipate change in the frequency of therapeutic sessions or therapeutic focus over the duration of the study  (14) have a clinically significant laboratory abnormality, in the opinion of one of the principal investigators or study physicians  (15) currently take medications that potentially limit the tTIS efficacy  (16) have a non-correctable clinically significant sensory impairment (i.e., cannot hear well enough to cooperate with an interview)  (17) have a clinical finding that is unstable or that, in the opinion of the investigator(s), would be negatively affected by the study medication or that would affect the study medication (e.g., diabetes mellitus, hypertension, unstable angina)  (18) have uncorrected hypothyroidism or hyperthyroidism. Subjects needing a thyroid hormone supplement to treat hypothyroidism will be excluded if they have NOT been on a stable dose of the medication for 30 days prior to enrolment  (19) have any other condition that, in the opinion of the investigator(s), would adversely affect the subject’s ability to complete the study or its measure  (20) wear a hairstyle or headdress at the time of the stimulation that prevents electrode contact with the scalp or would interfere with the stimulation (e.g., thick or curly hair)  (21) have any contraindications for receiving tTIS or undergoing MRI scans (e.g., hip circumference >180 cm or metal in the body)  **Justifications for any exclusions based on race, gender, or ethnicity**: non-English speaking individuals are excluded because the ability to accurately and completely communicate study information, answer questions about the study, and obtain consent are necessary |
